# Biology and Ecology of *Lygus pratensis* (Linn, 1758) (Heteroptera: Miridae): Towards the Practical Management of Cropping Landscapes in China

**DOI:** 10.3390/insects16050441

**Published:** 2025-04-23

**Authors:** Pengfei Li, Changqing Gou, Hongzu Feng

**Affiliations:** 1 College of Life Science and Technology, Tarim University, Alar 843300, China; 10757202064@stumail.taru.edu.cn; 2 Key Laboratory of Integrated Pest Management (IPM) of Xinjiang Production and Construction Corps in Southern Xinjiang, Tarim University, Alar 843300, China; 18109976026@163.com; 3 Scientific Observing and Experimental Station of Crop Pests in Alar, Ministry of Agriculture, Tarim University, Alar 843300, China; 4 The National and Local Joint Engineering Laboratory of High Efficiency and Superior-Quality Cultivation and Fruit Deep Processing Technology on Characteristic Fruit Trees, Tarim University, Alar 843300, China

**Keywords:** mirid bug, farmland landscape, ecological regulation, IPM

## Abstract

*Lygus pratensis* (Linnaeus) (Hemiptera: Miridae) is an agricultural pest widely distributed across Europe, China, North Africa, the Middle East, and India. The population of *L. pratensis* has increased in recent years due to the prolonged reproductive period, high productivity, and strong adaptability of adult *L. pratensis*, along with other factors such as changes in crop planting schemes. It significantly damages cotton production and adversely affects commercial crops such as alfalfa and fruit trees. Recent studies on the interrelationship between landscape features and pest management have provided new insights for controlling *L. pratensis*. This paper primarily reviews multiple aspects, including its life history and habits, host plants, pheromones, diapause characteristics, migratory dispersal, the relationship between *L. pratensis* occurrences and environmental factors, chemical control and resistance, sampling surveys and prevention indicators, ecological control, molecular genetic control, and the ecological effects of farmland landscape patterns on *L. pratensis*. We focus on the outlook for the conservation effectiveness of farmland landscape patterns on the diversity of natural enemies and the developmental direction of the ecological regulation of *L. pratensis*. The aim is to develop new control strategies and technologies to enhance the comprehensive control of *L. pratensis*.

## Simple Summary

*Lygus pratensis* (Linnaeus) (Hemiptera: Miridae) is a significant agricultural pest extensively documented in various countries and regions. This pest causes considerable damage to cotton production and can also adversely affect cash crops such as alfalfa and fruit trees. The infestation of *L. pratensis* is on the rise, and chemical control using broad-spectrum insecticides remains the primary method for managing *L. pratensis* in cotton fields, which has resulted in an increase in resistance to these agents in *L. pratensis* over the years. We primarily review multiple aspects, including its life history and habits, host plants, pheromones, diapause characteristics, migratory dispersal, the relationship between *L. pratensis* occurrences and environmental factors, chemical control and resistance, sampling surveys and prevention indicators, ecological control, molecular genetic control, and the ecological effects of farmland landscape patterns on *L. pratensis*. We focus on the outlook for the conservation effectiveness of farmland landscape patterns on the diversity of natural enemies and the developmental direction of the ecological regulation of *L. pratensis.* The aim is to develop new control strategies and technologies to enhance the comprehensive control of *L. pratensis*.

## 1. Introduction

Approximately 60% of Hemiptera insects are phytophagous and have more than 40,000 species [1,2]. Among these, Miridae represents one of the most diverse families within the order Hemiptera, encompassing 11,000 species across 1200 genera [3]. These pests have caused severe economic damage to crops in several countries [4,5,6]. Transgenic *Bacillus thuringiensis* (Bt) cotton has been planted on a large scale worldwide [7,8,9], leading to a gradual decline in the use of broad-spectrum insecticides. Consequently, mirid bugs (Hemiptera: Miridae) have emerged as a significant pest in cotton fields, prompted by a lack of effective management that has increased in their population. For example, mirid bugs have become important pests on Bt cotton in North America [4,10]. In China, research indicates a considerable rise in mirid bug populations following the cultivation of Bt cotton; with the ongoing expansion of the area of Bt cotton acreage, the damage inflicted by mirid bugs on cotton production in China is anticipated to increase progressively [5,11,12].

*Lygus pratensis* (Linnaeus, 1758) (Hemiptera: Miridae) is a significant agricultural pest that was reported for the first time in Europe and has been extensively documented in various countries and regions [13,14]. At present, 21 species of *Lygus* pests have been reported in the world, and 13 species have been found in China, among which *L. pratensis* has a wide host range, mostly sucking the flowers, stems, leaves, and young parts of plants and affecting the growth and development of the plants, although *L. pratensis* has been observed to feed on the eggs of *Aphis gossypii* (Glover) (Homoptera: Aphididae), *Helicoverpa armigera* (Hübner) (Lepidoptera: Noctuidae), and *Leptinotarsa decemlineata* (Say) (Coleoptera: Chrysomelidae) as well as the larvae [15]. However, this pest causes considerable damage to cotton production in China [16,17] and can also adversely affect cash crops such as alfalfa and fruit trees [18,19]. The infestation of *L. pratensis* is on the rise [20]. The growth and development of *L. pratensis* are categorised into three stages: egg, first to fifth instar nymph, and adult [21,22]. There is considerable overlap among generations [23]. Adults exhibit a prolonged reproductive period, high adaptability, significant reproductive potential, and strong flight dispersal capabilities [14,24,25], enabling them to transfer damage among various host plants in response to seasonal changes [26,27].

Field control of *L*. *pratensis* uses a combination of control methods, but chemical control using broad-spectrum insecticides remains the primary method for managing *L. pratensis* in cotton fields [28,29]. Traditional insecticides such as cypermethrin, imidacloprid, malathion, and thiamethoxam continue to be utilised in significant quantities to control *L. pratensis*. Resistance among field populations of *L. pratensis* in the seven farming-pastoral ecotones of northern China to beta-cypermethrin is increasing, with some populations exhibiting moderate cross-resistance to deltamethrin [30]. There is an urgent need to explore practical and sustainable methods as alternatives to the chemical management of *L. pratensis*. In recent years, research on the interrelationships between landscape features and pest management has garnered significant attention [31,32,33,34,35], providing new insights for controlling *L. pratensis*. Expanding crop habitat areas and reducing non-crop habitat areas can simplify farmland landscape structure, resulting in decreased biodiversity. This decline in biodiversity, in turn, affects the prevalence and damage caused by pests in farmland landscapes [36,37,38]. It has also been demonstrated that increased vegetation diversity in crop habitats enhances the population size of natural enemies of pests, thereby reducing or suppressing pests’ population size [39,40,41]. Different species of pests respond variably to different landscape variables. A comprehensive analysis of the correlation between landscape structure and pest population size and their ecological effects can aid in pest population regulation through habitat management strategies.

This paper thus focuses on the progress of research regarding the biology, ecology, and integrated management of *L. pratensis*. It discusses the potential for farmland landscape structure to regulate the population dynamics of *L. pratensis* and analyses the feasibility of incorporating the ecological regulation of pest populations by farmland landscape structure into integrated pest management.

## 2. Distribution and Damage

**Figure 1:**
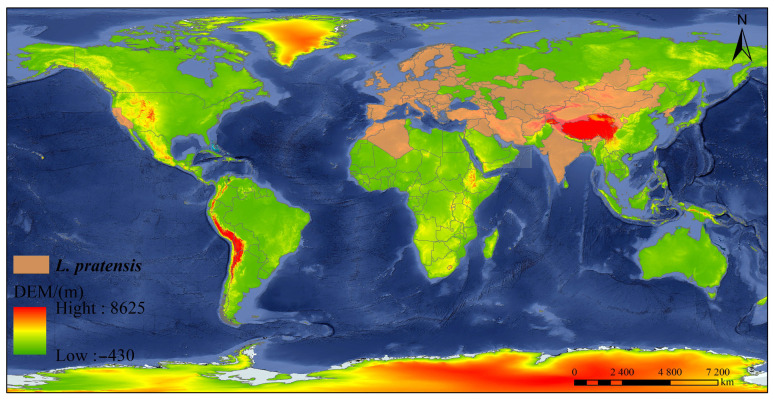
Worldwide distribution of *L. pratensis*. Map data © Google, © 2024. Image captured from Google Maps (https://code.earthengine.google.com/?project=ee-hshwywhejwjw7, accessed on 9 August 2024). Data were collected from articles published from 1860, when the first *L. pratensis* was described, to 2024 and cited in indexed journals from Google Scholar, Science Direct, PubMed, Scopus, Web of Science, MDPI, Taylor & Francis, etc.

*Lygus pratensis* is widely distributed across Europe, North Africa, the Middle East, and India [13,14,42] and has also been reported in countries such as Turkey and Uzbekistan (Figure 1) [43]. The grassland regions of Central Asia, Eastern Europe, and the Middle East are the earliest distribution areas of *L. pratensis*, exhibiting significant ecological adaptability in temperate and subtropical regions [44]. In the 1950s, *L. pratensis* was first documented in northwestern China [45]. With the expansion of agriculture, it gradually spread from natural habitats into agricultural ecosystems [46]. Cotton and alfalfa serve as important host plants for *L. pratensis*. These two crops are key economic plants throughout Xinjiang and the Hexi Corridor, eventually extending to central China [47]. The frequent trade of cotton and alfalfa may have increased the opportunities for the passive spread of *L. pratensis* in the western regions of China [14]. There is a possibility that *L. pratensis* was inadvertently introduced to North America from Europe in the mid-20th century through plant trade and human migration; however, it did not establish stable populations in the wild. Previous studies mentioning *L. pratensis* likely referred to *Lygus lineolaris* (Palisot de Beauvois) (Hemiptera: Miridae). Consequently, the presence of *L. pratensis* in North America remains to be confirmed [48].

In the 1950s and 1970s, cotton fields in Xinjiang were severely affected by *L. pratensis*, resulting in over 62% of cotton flower buds and bolls falling off [49,50,51]. In the 1980s to 1990s, to control the damaged caused by successive outbreaks of the cotton bollworm *A*. *gossypii* and *H*. *armigera* [52], a gradual increase in the frequency and volume of pesticide application in cotton fields and the large-scale use of new types of chemical pesticides were utilized; thus, *L. pratensis* was effectively managed and gradually evolved into a secondary pest of cotton [53]. In the 21st century, due to the adjustment of the crop cultivation structure in Xinjiang, particularly with the extensive cultivation of Bt insect-resistant cotton and urban development, *L. pratensis* has become rampant again, with its population increasing annually and the extent of damage further expanding [54,55,56]. *Lygus pratensis* has become the dominant species of mirid bugs in cotton fields in southern Xinjiang [57]. *Lygus pratensis* exhibits a wide range of feeding habits (Table 1); in addition to affecting cotton, it also feeds on legumes, grains, vegetables, and horticultural crops, particularly alfalfa, sugar beets, maize, tobacco, pumpkins, potatoes, hemp, sunflowers, grapes, cucumbers, cassis, strawberries, and many other cultivated plants, with 159 species of plants documented as hosts for *L. pratensis* (Table 1) [17,18,58,59,60,61,62,63,64,65,66]. Moreover, *L. pratensis* was found to damage the hemp hibiscus *Hibiscus cannabinus* (Linnaeus) (Malvales: Malvaceae) [67], establishing *L. pratensis* as an important pest of economically significant crops and ecologically vital grass pastures [14,68,69].

*Lygus pratensis*, comprising both nymphs and adults, feeds on various host plants’ young tissues and reproductive organs [69,70]. Nymphs feed on plants’ young shoots and flower buds, and adults can damage young leaves and fruits. There is a difference in preference for host plants between the adults and nymphs, with the nymphs unable to achieve eclosion after feeding on *Salsola collina* (Chenopodiaceae: Salsola), *Chenopodium Strictum* (Chenopodiaceae: Chenopodium), and *Solanum nigrum* (Solanaceae: Solanum), but the adults can feed normally, resulting in differing damage symptoms among different hosts. When infesting cotton, nymphs and adults primarily suck the young tissues, leading to the drying of buds and bolls as well as the shedding of flower buds and bolls, which may cause the loss of 62.2% to 82.2% of bolls in severe cases. In instances of less severe damage, the cotton peaches may become deformed or blackened, which can significantly diminish cotton quality, impact yield, and result in substantial economic losses in cotton production [71,72,73]. When the insect damages alfalfa, it typically congregates on young shoots, young stems, the undersides of leaves, and flower vessels, pricking and sucking sap from the tissues and thereby affecting plant development. This results in necrosis of the growing point, growth inhibition, crumpling and wilting of leaves, and withering of flower buds. In severe cases, the plant may wither and die, causing economic losses [61,69]. *Lygus pratensis* primarily damages fruit trees by targeting flower buds; excessive stinging results in halted development and bud drop, with young fruits being affected, leading to the appearance of black spots or small protrusions on the surface of the fruit as well as necrosis of the fruit flesh tissue. Most affected fruits fall off; if not prevented and controlled, this can cause a 10% to 30% yield loss [13,74]. Damage to *H*. *cannabinus* destroys the apical meristematic tissue and leads to secondary stems and leaf fragmentation [67]. *Lygus pratensis* is also a carrier of many plant pathogens, particularly potato leaf roll virus (PLRV), potato virus Y (PVY), potato virus A (PVA), potato virus S (PVS), and potato virus M (PVM). Once on the Pamir plateau, *L. pratensis* threatened the safe cultivation of potatoes [75,76,77] and is associated with alfalfa virus, bacterial bean blight, tobacco mosaic virus, and beet mosaic viruses [78]. Infestation by *L. pratensis* significantly reduces crop yield and quality [79], posing a serious threat to the safe production and sustainable development of the cotton and forest fruit industries [80,81].

**Table 1:** The taxonomic distribution of *Lygus pratensis* host plants.

Number	Family Name	Species Quantity	Proportion (%)
1	Amaranthaceae	22 [17,18,82]	13.8
2	Asteraceae	21 [17,18,27,82]	12.2
3	Fabaceae	19 [17,18,27,82,83]	11.9
4	Brassicaceae	15 [17,18,64]	9.4
5	Solanaceae	10 [17,18,82]	6.3
6	Rosaceae	9 [17,18]	5.7
7	Chenopodiaceae	7 [18,27,64,82]	4.4
8	Cucurbitaceae	7 [17,18]	4.4
9	Malvaceae	7 [17,18,64,82]	4.4
10	Poaceae	6 [17,18]	3.8
11	Apiaceae	5 [17,18]	3.1
12	Convolvulaceae	4 [17,18]	2.5
13	Polygonaceae	3 [17,18]	1.9
14	Labiatae	3 [17,18]	1.9
15	Plantaginaceae	1 [18]	0.6
16	Zygophyllaceae	1 [17]	0.6
17	Dioscoreaceae	1 [18]	0.6
18	Balsaminaceae	1 [17]	0.6
19	Elaeagnaceae	1 [17]	0.6
20	Juglandaceae	1 [17]	0.6
21	Euphorbiaceae	1 [17]	0.6
22	Nyctaginaceae	1 [17]	0.6
23	Berberidaceae	1 [18]	0.6
24	Portulacaceae	1 [17]	0.6
25	Rhamnaceae	1 [17]	0.6
26	Tamaricaceae	1 [18]	0.6
27	Apocynaceae	1 [17]	0.6
28	Cannabaceae	1 [17]	0.6
29	Oxalidaceae	1 [18]	0.6
30	Pedaliaceae	1 [17]	0.6
31	Moraceae	1 [18]	0.6
32	Ulmaceae	1 [18]	0.6
33	Vitaceae	1 [18]	0.6
34	Oleaceae	1 [18]	0.6
35	Linaceae	1 [18]	0.6

## 3. Biosystematics

### 3.1. Morphology

**Figure 2:**
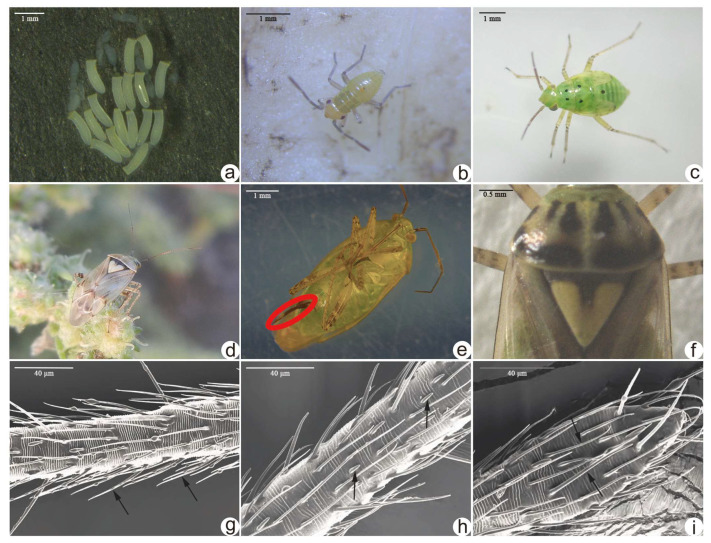
Morphology of the various stages of *Lygus pratensis*. (**a**) Eggs are obtained by dissecting the abdomen of the female. (**b**) Nymph when newly hatched. (**c**) Second instar nymph. (**d**) Adult. (**e**) Ovipositor of the female. (**f**) Pronotum and scutellum. (**g**) Sensillum chaeticum. (**h**) Sensillum basiconicum. (**i**) Sensillum trichodeum. The red elliptical indicates the ovipositor, arrow indicates the Sensillum.

*L. pratensis* differs very little in size from *Lygus gemellatus* (Herrich-Schaeffer) (Hemiptera: Miridae) and *Lygus punctatus* (Zetterstedt) (Hemiptera: Miridae) and is easily confused in size. However, the body colour and some markings of *L. pratensis* differ considerably from the other species, and identification is relatively easy [84]. *Lygus pratensis* has three insect stages: egg, nymph, and adult (Figure 2a–d). Adult males measure 5.1–7.0 mm in length and 2.5–2.8 mm in width, while adult females range from 5.1 to 6.6 mm in length and from 2.4 to 2.7 mm in width, with antennae shorter than the body; the total antennal length of adult females was 1961.52 μm, and that of adult males was 1951.07 μm, which bear three types of sensilla: sensillum chaeticum, sensillum basiconicum, and sensillum trichodeum (Figure 2g–i) [85]. Males can be separated from other species in the small lobe of vesica without teeth on the right margin; additionally, its spicule is apically widened with teeth. Punctures on the middle part of the hemelytron are placed evenly, with the distance between them equal to or less than the puncture diameter; the anterior half of the clavus has some punctures removed from each other at a distance longer than the puncture diameter; punctures on the posterior half of the clavus are placed evenly and close to each other but distinctly separated from each other; the hemelytron has dense and short setae, often appearing more or less shiny [84]. The colour is yellowish green; the body colour of adult pasture *L. pratensis* changes during the overwintering period: from the non-wintering period to the early, middle, and late overwintering period, the body colour changes from yellowish green to greyish green, brownish brown, brownish red, and greyish brown in turn; the colour of the triangular-shaped scutellum also undergoes a series of changes with it, from bright yellow to yellowish green, yellow mixed with uneven blood colour, and greyish yellow in turn, and the central black spot of the base gradually becomes bigger; the colour of the dorsal plate of the prothorax changes from yellowish to greyish yellow, grey, black, and greyish black [86]. The incised pronotum resembles an orange peel; the lateral margins are black, featuring two black striae on the posterior margin and four longitudinal stripes in the centre, while the scutellum is yellow with a central blackish-brown indentation displaying a heart-shaped stripe (Figure 2f). Eggs are approximately 1.1 mm long and 0.3 mm in width, characterised by an inwardly curved stalk at the margin of the egg cover, which is lower in the centre than at the margin (Figure 2a). Newly hatched larvae are yellowish green, turning green in the fifth instar and measuring 3.5–3.7 mm in length and 1.7–1.9 mm in width, with one black dot on each side of the dorsal plate of the prothorax and each side of the lesser peltate; there is a black, rounded opening of the odorous gland on the posterior margin of the third abdominal segment, forming five black dots on the dorsal side of the body (Figure 2b–c) [23,84]. Females possess an ovipositor extending from their abdomens (Figure 2e).

### 3.2. Life History

**Figure 3:**
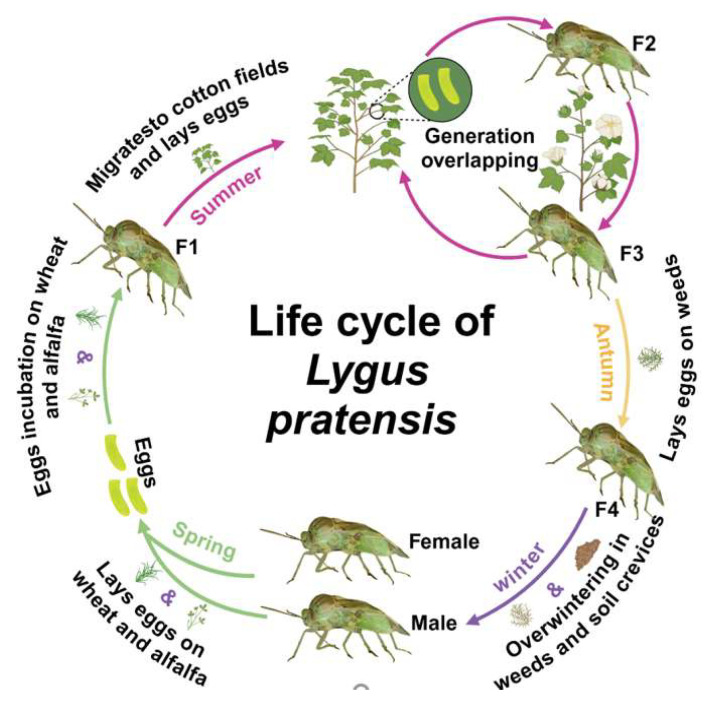
Life cycle of *Lygus pratensis* in southern Xinjiang, China.

In northern Russia, *L. pratensis* has one generation per year; in the southern regions, it can have up to four generations [78]. There is one generation per year in southern Finland [58], three generations per year in Kazakhstan [75], and four generations per year in Spain [87]. Additionally, four generations occur within one year in southern Xinjiang, China [14,17,88]. The response of insects to changes in environmental temperature is more evident than that of other organisms. The growth and development rate of insects is accelerated by temperature rise: the reproductive generation is increased, the period of occurrence is advanced, and the distribution and overwintering range is expanded, which may be the reason for the different number of generations of *L. pratensis* in different areas [89]. *Lygus pratensis* overwinters as dormant adults under weeds, decayed material, and debris near cotton fields. The following spring, the host emerges from hibernation following germination and concentrates in wheat fields, alfalfa fields, and early spring nectar plants such as apple, pear, peach, and apricot orchards. It prefers to sting and extract sap from young leaves, stems, and buds and begins mating and laying eggs after feeding for a period. It lays more eggs on the young stems, petioles, veins, or buds, where the first generation of *L. pratensis* develops. In June, the first-generation adults migrate to cotton fields, producing the second generation. Individuals of the second and third generations develop on cotton plants, and the population reaches its maximum density, infesting cotton and causing bud and boll shedding [57,90]. In early September, as the cotton plant matures and becomes unsuitable for *L. pratensis* to feed on, adults of the third generation migrate away from the cotton field, primarily laying eggs on quinoa and daisy family weeds to reproduce the fourth generation (Figure 3, Table 2) [13]. Adult *L. pratensis* possesses strong flight capabilities [91] and continues to transfer and spread between hosts, complicating control efforts.

Diapause is a crucial adaptive strategy insects employ to withstand adversities such as food shortages, low temperatures, and inadequate photoperiods. It also plays a vital role in synchronising the developmental stages of insects with their external environment [92,93]. In southern Xinjiang, *L. pratensis* commences its stagnation in mid- to late October each year, with changes in body colour and the development of the reproductive system serving as important criteria for determining whether *L. pratensis* is in a state of diapause. Temperature and photoperiod are the primary factors influencing the induction and termination of diapause in *L. pratensis*. Temperature regulates the metabolic rate, growth, and development of *L. pratensis*, significantly influencing the timing of diapause termination. Specifically, low temperatures lead to the cessation of reproductive development. Furthermore, photoperiod plays a crucial role in *L. pratensis’s* ability to perceive seasonal changes and modulate the diapause cycle, determining whether or not to enter a diapause state. *Lygus pratensis* was found unable to lay eggs under various photoperiod conditions at 12 °C. The egg-laying rate and quantity increased with the extension of the photoperiod at 16 °C, which aids in understanding the adaptive responses of *L. pratensis* to its ecosystem and provides a foundation for improved monitoring of *L. pratensis* population dynamics, thereby ensuring timely control measures [22,94]. The photoperiod is a determinant of the diapause termination rate in *L. pratensis*. A prolonged photoperiod favours the termination of diapause. An increase in temperature does not influence the rate of diapause termination, but it can reduce the time needed to terminate diapause [95]. Adult *L. pratensis* could successfully overwinter soil moisture content, ranging from 5% to 40%. The overwintering survival rate showed an increasing and then decreasing trend with the increase in soil moisture content, with the highest survival rate when the soil moisture content was 15% and the lowest survival rate when the soil moisture content was 40% [96]. In conjunction with the relationship between the occurrence of *L. pratensis* diapause and environmental factors, winter irrigation can be carried out during the diapause period of *L. pratensis* to reduce the overwintering base of *L. pratensis*; the duration of the diapause period of *L. pratensis* can also be predicted so that precise spraying of insecticides or the introduction of natural enemies can be adopted soon after the end of the diapause period when the adults emerge, thereby reducing the number of the population of the overwintering adult *L. pratensis*.

### 3.3. Migration and Dispersion

Research demonstrated that the flight distance, flight time, and flight rate were at their highest in 10-day-old adults of *L. pratensis*. The flight capability of *L. pratensis* feeding on pear blossoms after hibernation was the strongest, while that of *L. pratensis* feeding on wheat was the weakest. The host plant provides more energy for flight and nutrients for the growth and development of *L. pratensis*. The flowers of plants contain many nutrients, and soluble sugars are an important component of flower buds and are present in high levels, which is likely the main reason why pear blossoms provide more energy material for *L. pratensis* to use for flight. In the mated state of *L. pratensis*, the average flight distance (19.5 km) and average flight time (6.9 h) of females were significantly greater than those of males. In contrast, the difference between unmated females and males was not significant. After mating, adult females have an increased ability to fly in search of new host plants to lay their eggs, which may explain why females fly greater distances than males. Temperature significantly affected the flight ability of *L. pratensis*, with the greatest flight ability observed at 24 °C; the flight ability was weakest at low temperatures of 16 °C or high temperatures of 36 °C. Humidity influenced the flight distance and flight time of *L. pratensis* but had no significant effect on flight speed; the total flight distance and flight time were longest when the relative humidity was 75% and shortest when it was 30%. Darkness significantly reduced the total flight distance and flight time of *L. pratensis*, although different light intensities did not significantly affect flight ability [91]. In southern Xinjiang, the overwintering adults of *L. pratensis* commence their activities from late March to early April, primarily feeding on alfalfa, rapeseed, and various weeds. They complete their first generation on these host plants; thus, it is essential to focus on controlling *L. pratensis* in alfalfa and rape fields in the spring to minimize *L. pratensis* density. The first-generation adults gradually migrate and spread into the cotton field from the end of May to mid-June. In the migration process, they choose to feed on the nearby sunflower, safflower, and other weeds, and this is a small-distance migration, with a maximum migration distance of 750 m. Weeds around the cotton fields play a significant transitional role in the migration of *L. pratensis* into the cotton fields. Therefore, eradicating these weeds and planting sunflower and safflower as trap crops around the cotton fields can create an unfavourable environment for the migration of *L. pratensis*, effectively controlling their presence in cotton crops. *L. pratensis* also reproduces and causes damage within cotton fields. Following the completion of their second and third generations, the third generation of adults gradually migrate from the cotton fields to feed on weeds in late August, when they complete their fourth generation. The third generation of *L. pratensis* vacates the cotton fields, predominantly focusing on quinoa host plants. Consequently, implementing prevention and control measures for *L. pratensis* in Chenopodiaceae host plants in the autumn can significantly reduce the overwintering population of *L. pratensis* and the incidence of infestations in the following year. The fourth generation of adults overwinters in the stubble of their host plants, including weeds and the decaying leaves in the fields, during the latter half of October [17,27].

## 4. Ecology

### 4.1. The Occurrence of L. pratensis Is Related to the Environment

**Figure 4:**
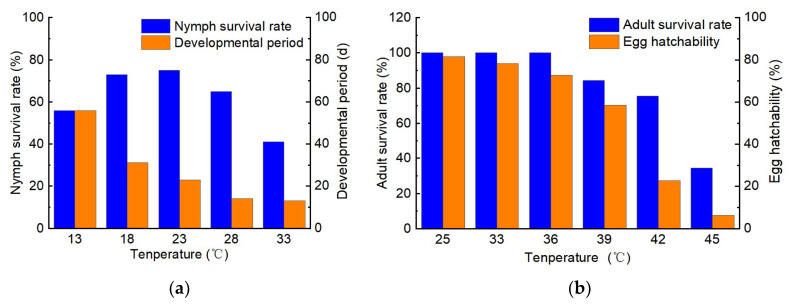
The effect of temperature on *Lygus pratensis*. (**a**) Nymph. (**b**) Adult [94,96].

Insects have developed specialised ecological adaptations to external environmental factors such as temperature, photoperiod, and moisture through extensive evolutionary processes. Among these factors, temperature, photoperiod, and humidity are the most critical ecological determinants influencing the synchronised growth, development, and reproduction of insects and their host plants. Temperature directly impacts the growth, development, and reproduction of individual insects and can influence the growth and distribution of their populations [97]. Loamy soils that are generally wetter, cooler, and contain more sand are best suited for overwintering *L. pratensis*. The soil moisture of the overwintering sites of *L. pratensis* significantly affects their overwintering survival rates. Suitable overwintering sites exhibit soil moisture contents ranging from 5.0% to 38.7%, with the highest survival rate of 28.9% was achieved at 15% soil moisture content. Variations in food resources across different environments influence the overwintering survival of *L. pratensis*, with food-providing adults showing a survival rate of up to 33.6% during overwintering, which is 7% higher than that of adults without food [96]. Rearing *L. pratensis* at temperatures between 15 °C and 30 °C resulted in a shorter developmental duration and a higher survival rate, as evidenced by the observation that the developmental durations of both eggs and nymphs decrease with rising temperatures; however, excessively high (33 °C) or excessively low (13 °C) temperatures adversely impact the growth and development of *L. pratensis* [22]. When temperatures are too low, the developmental duration of each life stage of *L. pratensis* is prolonged. Eggs do not hatch at temperatures below 10 °C, and newly hatched nymphs fail to develop normally; survival rates are low, and adults do not lay eggs. Conversely, excessively high temperatures significantly impair the growth and development of *L. pratensis*, with 37.4 °C being lethal to nymphs and 40.9 °C resulting in the death of eggs. At the same time, adult egg-laying behaviour is typically inhibited (Figure 4a) [13,94]. Short-term exposure to high temperatures (42 °C for 2 h) significantly reduces egg hatchability and adult survival (Figure 4b) [97]. Long photoperiods at suitable temperatures enhance the egg-laying rate of *L. pratensis* [95]. *Lygus pratensis* prefers warm and humid environments; following irrigation, the relative humidity in cotton fields increases, leading to a significant rise in *L. pratensis* migrating into these fields, resulting in severe damage to the cotton [98]. Intercropping fruit with cotton specifically impacts the population dynamics of *L. pratensis* in cotton fields, which mitigates its proliferation and damage to these environments [99].

### 4.2. Influence of Plant Volatiles on L. pratensis

**Figure 5:**
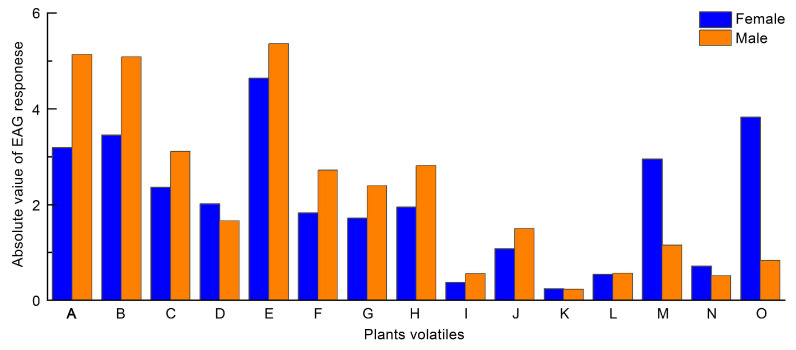
EAG responses of *Lygus pratensis* to host plant volatiles. (A) α-Pinene. (B) 1,6,10-Dodecatriene, 7,11-dimethyl-3-methylene, (*E*)-. (C) 1-Caryophyllene. (D) 3-Hexen-1-ol, acetate, (*Z*)-. (E) Nonanal. (F) Dibutyl phthalate. (G) 1-Hexanol, 2-ethyl. (H) Butylated hydroxytoluene. (I) 1(3H)-Isobenzofuranone, 6-(dimethylamino)-3,3-bis [4-(dimethylamino) phenyl]. (J) Methyl salicylate. (K) Linalool. (L) 2-Hexen-1-ol, (*E*)-. (M) Ocimene. (N) Isothiocyanic acid sec-butyl ester. (O) Phenylacetaldehyde [19,64].

Plant volatiles were found to greatly influence host selection, mating, egg laying, feeding, and developmental behaviour of phytophagous insects [100]. Sun et al. [19] identified 31 volatile compounds from seven host plants. The relative values of electroantennography (EAG) responses of female and male bugs to different plant volatiles were significantly different, with the relative values of the responses of female bugs to the same compounds being greater than those of male bugs. In the determination of chemotaxis, *L. pratensis* females exhibited significant differences in behavioural responses to most concentrations of nonanal; 1,6,10-dodecatriene,7,11-dimethyl-3-methylene (*E*)-; α-pinene; 1-caryophyllene; 3-hexen-1-ol, acetate, (*Z*)-; and 1-hexanol, 2-ethyl. In contrast, males showed significant differences in behavioural responses to only certain concentrations of α-pinene and nonanal (Figure 5). This phenomenon may be due to the different roles played by male and female *L. pratensis* in behaviours such as finding hosts and reproducing offspring. Also, the antenna of *L. pratensis* may have some differences in odour perception depending on sex. Xia et al. [64] identified a total of 25 volatiles in five species of *L. pratensis* host plants, with the highest relative EAG response value for *L. pratensis* females recorded for phenylacetaldehyde. The chemotactic responses to phenylacetaldehyde and isothiocyanic acid sec-butyl ester reached highly significant and significant levels of attraction to *L. pratensis* females, respectively (Figure 5); both of these compounds are present in *B. napus* (Table 3). This resulted in host plants existing under the same spatial conditions, with *L. pratensis* showing a preference for feeding on *B. napus*. It is thus concluded that oilseed rape is a very good *L. pratensis* attractant plant, and phenylacetaldehyde, along with isothiocyanic acid sec-butyl ester, may serve as potential elicitor candidates alone. These compounds could be designed as modifiers of *L. pratensis* behaviour for the detection and control of *L. pratensis*, aligning with the principles of sustainable plant protection.

### 4.3. Impact of Agricultural Landscapes

Crop habitats are the primary environments for farmland pests, and their diversity is a crucial factor in agroecosystems, serving as a measure of landscape structure [101,102]. Multiple crop habitats offer a variety of host plants for pest populations, making food and shelter more readily available for certain pests [103,104]. Non-crop habitats within agroecosystems are essential components of the agroecosystem landscape, playing a significant role in maintaining ecosystem stability and securing ecosystem services. These habitats are vital for the reproduction and development of pests, primarily forage and breeding in crop habitats while utilising non-crop habitats as alternative foraging and breeding sites [105,106]. It was observed that expanding the area planted with date palms around cotton fields enhanced the population of second-generation *Apolygus lucorum* (Meyer–Dür) (Hemiptera: Miridae) within these fields. Conversely, an increase in the extent of roads and shrub strips was associated with a decrease in the population density of third-generation *A. lucorum* in cotton fields. Landscape features such as water bodies and roads can act as barriers, disrupting the migratory movements of *A. lucorum* [107,108]. Furthermore, increasing the area planted with maize around cotton fields led to a rise in the population of third-generation *A. lucorum* within these fields [109]. The landscape pattern of farmland—particularly the area share, shape, and spatial arrangement of various types of habitats—has a significant regulatory effect on the population size of adult cotton field *L. pratensis* [110]. Planting non-host plants, such as maize, around the cotton field as an isolation zone can inhibit the migration of *L. pratensis* into the cotton field. Furthermore, maize can attract various natural enemies of *L. pratensis*, thereby protecting the cotton. Reasonable planning of field size is needed to avoid the cotton field being too large, resulting in the rapid spread of *L. pratensis* once it occurs. Adopting a more regular shape for cotton fields can decrease the ratio of field edge to area. Since *L*. *pratensis* typically gathers and invades at the edges of farmland, a regular shape can mitigate the edge effect and reduce the pathways for invasion by *L. pratensis*. It is of considerable theoretical and practical significance to systematically investigate the correlation between landscape factors and *L. pratensis* populations at the scale of farmland landscapes and to elucidate the ecological impacts of farmland landscape structure on *L. pratensis*. This understanding may facilitate regional environmental management of the pest by adjusting crop layouts, a topic that has received relatively limited attention thus far.

## 5. Prevention and Control

### 5.1. Sampling Survey

*L. pratensis* eggs are laid in plant tissues, with only the egg cover visible on the surface, primarily located in the upper part of cotton plants. *L. pratensis* is relatively small in size during the nymph stage, flexible, easily startled, and tends to hide during the day, primarily found on leaves and buds. The flight ability of the adults is greatly enhanced, allowing them to seek refuge in the shade of weeds and the lower parts of plants during high temperatures, where they are predominantly located on the leaves [111,112]. Because of the above special habits, it is not easy to investigate the population of *L. pratensis*. The stain-counting method was employed in one study for an egg survey. Eosin Y, the staining agent, was prepared as a 1% solution using 75% alcohol, and this solution was stored at 4 °C for later use. Then, 25 mL of the 1% Eosin Y solution was added to a beaker, and live plants were collected from the field, with various tissues disintegrated, including branches and leaves. The plant tissues were individually immersed in the solution for 2 min, after which they were removed and rinsed with running water, and the surface droplets were dried using absorbent paper. The egg of *L. pratensis*, which was present in the plant tissues, became stained red, creating a distinct contrast with the colour of the plant tissues. The stained plant tissue was directly observed and counted using a microscope or a hand-held magnifier. This method allowed for the accurate enumeration of *L. pratensis* eggs on living plants; however, it involved considerable work [57,113]. For the nymph survey, visual inspection was conducted on sparsely planted crops such as cotton and fruit trees, while the plant-flapping method was utilised for densely planted host plants like alfalfa and weeds. A white porcelain basin measuring 30 cm x 40 cm was positioned at the lower part of the plant. *L. pratensis* was collected by tapping the middle and upper sections of the plant, causing *L. pratensis* to fall into the basin, where a rapid count was conducted.

Simultaneously, the area of the site containing the investigated plants was recorded during each survey to estimate the population density of *L. pratensis* per unit area [114,115]. The adult survey methods included visual inspection, plant-flapping and net-sweeping methods, colour sticky boards, and sex lure trapping. Investigators may have possessed varying degrees of recognition abilities concerning *L. pratensis*. Furthermore, the activity intensity of *L. pratensis* fluctuates at different times of the day; thus, conducting investigations at varied times may have influenced the results owing to the differing activity states of *L. pratensis*. The colour sticky board method and the sex lure technique are influenced by the flight capabilities and sexual response abilities of *L. pratensis*. Some individuals may exhibit limitations in flight ability or a reduced sensitivity to sex lures due to their physiological state, environmental factors, or other variables. Additionally, the volatilisation and diffusion of sex lures are impacted by environmental conditions such as wind, temperature, and humidity, which consequently affect the trapping efficacy. When establishing sticky boards and sex traps, aspects such as the location, height, and quantity of placements can also influence the outcomes of the survey. Zhang et al. [73] reported that the net-sweeping method can more accurately reflect the number of adult *L. pratensis*. Due to the overlapping generations of *L. pratensis*, adults and nymphs co-occur, making it common to use net-sweeping and plant-flapping methods to investigate adults and nymphs simultaneously. The combination of the net-sweeping and plant-flapping methods enables a rapid and precise assessment of the field’s population size and distribution of *L. pratensis* [116]. A comparison of the survey results with the established control thresholds indicates that if the survey results are significantly lower than these thresholds, the intensity or frequency of preventive measures can be appropriately reduced to prevent excessive interventions, thereby avoiding resource wastage and environmental pollution. When the population of *L. pratensis* is low and does not reach the prevention and control thresholds, the primary focus should be on agricultural control measures, such as removing weeds and implementing optimal planting densities. Conversely, if the population density of *L. pratensis* as determined by the survey approaches or exceeds the prevention and control thresholds, this suggests that the damage caused by *L. pratensis* may result in substantial crop losses. In such cases, it is essential to select appropriate chemical control agents or promptly release the insects’ natural enemies for chemical and biological control measures.

### 5.2. Prevention and Control Indicators

With the extensive cultivation of insect-resistant cotton in Xinjiang, the population of *L. pratensis* has increased significantly, leading to exacerbated damage, highlighting the need to clarify the control indices for each reproductive period. Wang et al. [72] investigated the relationship between the population of *L. pratensis* and cotton yield loss across different growth stages of cotton. They discovered that *L. pratensis* infestation during the bud stage had the most significant impact on cotton yield loss, followed by infestation at the flowering and bolling stages. Control thresholds for *L. pratensis* were established based on the economic threshold for cotton, which was determined to be 12 heads per 100 plants at the bud stage, 20 heads per 100 plants at the flowering stage, and 41 heads per 100 plants at the bolling stage, respectively. These control thresholds and the net-sweeping and plant-flapping methods for assessing *L. pratensis* are straightforward and practical, facilitating the rapid identification of the critical control period for *L. pratensis*.

### 5.3. Ecological Control

To mitigate the damage caused by *L. pratensis*, farmland surrounding alfalfa should be converted to food crops or cash crops whenever possible [117]. This establishes an isolation zone when the overwintering adults of *L. pratensis* begin to emerge from late March to early April each spring, which can impede the transfer and spread of *L. pratensis* between different fields. Consequently, this limitation reduces the suitable feeding range of *L. pratensis*, thereby inhibiting their survival and reproduction, ultimately decreasing their population size. In fruit tree orchards, such as pear orchards, intercropping with cotton, alfalfa, vegetables, and other crops that easily attract *L. pratensis* should be avoided when overwintering adults of *L. pratensis* begin to lay eggs in large numbers on alfalfa in early April; thus, the risk of fruit trees being oviposited by *L. pratensis* can be reduced [81]. Populations of *L. pratensis* on oilseed rape planted at the borders of cotton fields were significantly larger than those found in cotton fields adjacent to oilseed rape as well as in cotton fields without oilseed rape. The overall population of *L. pratensis* in cotton fields near oilseed rape was markedly reduced. The population of *L. pratensis* showed a gradual decline with increasing distance from the oilseed rape planting. Furthermore, the oilseed rape trapping zone can effectively capture *L. pratensis* while simultaneously providing a habitat for the growth and development of natural enemies, thereby promoting the conservation of these natural enemies in early cotton fields. Consequently, during the peak period of *L. pratensis* migration into cotton fields from early to mid-June, planting strips of oilseed rape environs in cotton fields not only exerts a significant trapping effect on *L. pratensis*; additionally, this approach has protective and proliferative effects on natural enemies in cotton fields, establishing oilseed rape as an ideal trapping plant for *L. pratensis* [64,118,119]. Zhang et al. [120] demonstrated that planting sunflowers around cotton fields in early April effectively diminished the number of *L. pratensis* in those fields. Moreover, Wang et al. [71] assessed the potential of safflower as a trap plant for controlling *L. pratensis*. They discovered that early-sown safflower hosted a greater number of *L. pratensis* compared to mid-sown or late-sown safflower. From mid-June to mid-July, the population of *L. pratensis* on safflower was significantly higher than that on adjacent cotton, indicating that safflower had a strong trapping effect on *L. pratensis*, which persisted for 4–5 weeks before gradually diminishing. Regarding cropping patterns, intercropping safflower as a trap crop proved more effective in reducing the densities of *L. pratensis* in cotton compared to the deployment of safflower as a “spot” trap crop or as a peripheral trap.

In recent years, North America and Europe have utilised the combination of farmland habitat management and regional landscape design to implement ecological pest control at multiple scales. The core principle involves the optimal integration of crop layout and agricultural management based on large-scale landscape design through the spatial configuration of agricultural landscape patterns, adjusting planting patterns and management techniques, and even altering the spatial configuration of agricultural landscapes to disrupt the life cycles of pest populations, thereby establishing and restoring the resources and pathways for natural enemy populations and ultimately maximising the agroecosystem’s pest control functionality [121,122,123,124]. This concept can be applied to the ecological regulation of *L. pratensis*. The control effect of landscape variables on the number of second-generation adults in the cotton field gradually weakened with the scale increase. There was a highly significant positive correlation between the population size of second-generation adults and the area ratio of buildings at the 500 m scale and the area ratio of forest belts and desolate sand habitats at the 1500 m scale. Conversely, a strong and highly significant negative correlation was observed between the population size of second-generation adults and the area ratio of the host plants at the 1000 m scale. Additionally, a strong and highly significant positive correlation was found between the population size of third-generation adults of *L. pratensis* and the area ratio of buildings at the 500 m scale. The population size of third-generation adults exhibited a negative correlation with the area ratio of the host plants, other crops, and water at the 1000 m scale. In comparison, it positively correlated with the perimeter area ratio at the 1500 m scale [110].

### 5.4. Physical Control

Jiang et al. [125] examined the phototropic response of the mirid to different wavelengths of lamps in southern Xinjiang. They discovered that a trapping lamp with a wavelength of 572 nm effectively trapped and killed *L. pratensis*, followed by trapping lamps with wavelengths of 418 nm and blacklight. Utilising the phototropism of *L. pratensis*, high-pressure mercury lamps, blacklight lamps, or frequency vibration insecticidal lamps were suspended around the cotton fields at a height of 2.0–2.5 m [126]. High-pressure mercury lamps and blacklight lamps emit ultraviolet light waves to attract *L. pratensis*, which are subsequently electrocuted by the electric grid surrounding the lamp upon approaching the light. Frequency vibration insecticidal lamps disrupt the physiological processes of *L. pratensis* by emitting vibrations at a specific frequency, potentially prolonging the activity cycle of *L. pratensis*. Moreover, the lamp casing is yellow, and when the lamp is activated at night, the light appears yellowish-green, attracting a significant number of *L. pratensis*, which are then electrocuted by the surrounding electric grid. It was observed that a single blacklight lamp could trap up to 60 *L. pratensis* within one day, demonstrating significant potential for reducing the population density of *L. pratensis* across the ecosystem when employed on a large scale [125].

Using sticky boards to trap pests is an environmentally friendly control tool based on the principle of insect colour tropism. The selectivity of *L. pratensis* towards host plants varies with seasonal changes [27,127], and their strong preference for a specific colour of sticky boards is related to the colour of the host plants they choose to feed on. Legrand and Los [128] discovered that *L. pratensis* in peach orchards exhibited a stronger attraction to pink sticky boards that closely matched the colour of peach petals. Tan et al. [83] investigated the trapping efficacy of eight types of coloured sticky boards and hanging methods on *L. pratensis* in alfalfa fields, finding that green sticky boards were the most effective for trapping *L. pratensis* when positioned at a height of 50 cm. Dilinu’er et al. [129] examined the trapping efficiency of various coloured sticky boards on *L. pratensis* in cotton fields. The found yellow sticky boards were the most effective, followed by purple sticky boards, while white sticky boards demonstrated the least efficacy. These studies comprehensively evaluated the feasibility of using sticky boards to control *L. pratensis*, optimised the trapping technology employing sticky boards, and provided theoretical and practical insights for the environmentally friendly prevention and control of *L. pratensis*.

### 5.5. Agricultural Prevention and Control

The timing of *L. pratensis* migration into the cotton fields was closely linked to the onset of the first irrigation and the growth of the cotton plants. Damage was particularly severe in cotton fields irrigated too early or excessively flooded [130]. The implementation of drip irrigation as opposed to flood irrigation, along with the application of N-P_2_O_5_-K_2_O at rates of 250–100–50 kg/hm^2^, effectively prevented the delayed ripening of cotton and, in combination with organic and chemical fertilizers, promoted the use of bio-fertilizers and microbial fungal fertilizers, which prevented cotton from being overly harmed by *L. pratensis* because it was too tender while also ensuring that the highest yields were obtained [118]. Removing weeds, dead branches, and leaves in and around cotton fields, especially from alfalfa and other overwintering host plants of *L. pratensis*, reduces the overwintering sites of the pests, effectively lowering the population of *L. pratensis* in the following year [131]. Concentrating on clearing weeds from roadsides and the ground before egg hatching and implementing management practices such as autumn ploughing and winter flooding further disrupts the overwintering environment of the pests. This reduces the overwintering population, effectively controlling *L. pratensis* [117].

### 5.6. Chemical Control

In recent years, the intensive cultivation of alfalfa and the increasing diversity of crops in northern China has resulted in a widespread occurrence of *L. pratensis* [132,133,134]. Concurrently, adult *L. pratensis* is highly active, has a broad array of host plants, exhibits varying levels of selectivity and preference for different hosts, and demonstrates seasonal host-switching behaviours, concealment, and significant mobility, which complicates *L. pratensis* control efforts [18,135]. Farmers and ranchers employ numerous integrated pest management (IPM) approaches [129]. Although yellow sticky traps have been effective in capturing *L. pratensis* in cotton fields and apple orchards [136], control of *L. pratensis* still primarily relies on chemical pesticides, which mainly consist of widely used insecticides such as phenylpyrazoles, pyrethroids, organochlorines, organophosphates, and carbamates [29,30,90,133,137]. Due to their low cost, high efficacy, and broad-spectrum activity, traditional insecticides such as cypermethrin, imidacloprid, acetamiprid, and thiamethoxam are frequently employed (Table 4) [69,136,138], leading to an increase in resistance among *L. pratensis* populations over the years. Tan et al. [30] conducted an indoor screening of *L. pratensis* indoors using high-efficacy cyfluthrin for 14 consecutive generations, increasing resistance to 42.555 times. Consequently, the frequency of high-efficacy cyfluthrin use should be reduced in the field.

Furthermore, *L. pratensis* exhibits varying degrees of resistance to phoxim, methomyl, cyhalothrin, imidacloprid, and abamectin; notably, it is at the highest risk of developing resistance to cyhalothrin in the field. Successive monitoring from 2015 to 2019 revealed that the resistance levels of the *L. pratensis* population in the field to phoxim, methomyl, and abamectin remained low. In contrast, resistance levels to cyhalothrin exhibited an upward trend. Zhu and Luttrell [139] treated two populations of imidacloprid-resistant *L. pratensis* with imidacloprid, recording survival rates of 94% and 38%, respectively, which were significantly higher than those of the sensitive lines. They also noted that some regional populations exhibited lower susceptibility to imidacloprid than the sensitive lines. In contrast, others exhibited resistance levels 1.6 to 5.9 times greater than those of the laboratory lines [140]. Da et al. [141] evaluated the toxicity of six insecticides against fourth instar larvae of *L. pratensis*, identifying abamectin as the most virulent, followed by permethrin and chlorpyrifos. Neonicotinoid insecticides effectively control pests (e.g., mirids, aphids, etc.) that use piercing-sucking mouthparts, as they can permeate plants through their roots, stems, and leaves [142,143]. The new generation of neonicotinoid insecticides, flupyradifurone, features a novel chemical structure with an LC_50_ value (48 h) for *L. pratensis* of 43.34 ng/cm^2^ and demonstrates minimal cross-resistance with other new nicotinic insecticides. Additionally, after a 30% reduction in flupyradifurone, it can be combined with green-peel orange oil and agricultural organosilicon additives to enhance its efficacy in preventing *L. pratensis* in pastures [144]. Therefore, phoxim, methomyl, abamectin, and flupyradifurone can be preferred agents for the chemical control of *L. pratensis*. In field applications, *L. pratensis* demonstrates relatively low resistance to flupyradifurone, and given that this insecticide exhibits low toxicity to bees, it can be applied at a rate of 114.75 g a.i./ha during the flowering stage of cotton to control *L. pratensis*, achieving a control efficacy of up to 89%. During the bud stage, when the damage inflicted by *L. pratensis* has the most significant impact on cotton yield, methomyl, which exhibits the least resistance, can be employed to rapidly reduce the *L. pratensis* population to below 12 heads per 100 plants, thus preventing substantial yield loss. In the boll stage, when cotton displays enhanced tolerance to *L. pratensis* damage, insecticides with relatively lower resistance, such as phoxim and abamectin, can be utilised. At this stage, maintaining *L. pratensis* densities below 41 heads per 100 plants is adequate to avert economic loss [72,144,145]. These agents used alternately in the field can significantly reduce the likelihood of *L. pratensis* developing resistance to chemical insecticides and enhance the effectiveness of control measures.

**Table 4:** LC_50_ of different pesticides against *Lygus pratensis*.

Pesticide Name	Pesticide Type	LC_50_ (ng/cm^2^)
Phoxim	Oganophosphorus	34.61 [146]
Methomyl	Carbamatepesticide	12.64 [146]
Imidacloprid	Neonicotinoid	45.61 [146]
High-efficacy cyfluthrin	Pyrethroids	55.85 [147]
Chlorpyrifos	Oganophosphorus	54.15 [141]
Abamectin	Agricultural antibiotic	40.96 [141]
Thiamethoxam	Neonicotinoid	196.48 [141]
Acetamiprid	Neonicotinoid	178.05 [141]
Flupyradifurone	Neonicotinoid	43.34 [144]

### 5.7. Biological Control

Parasitic wasps and spiders play a significant role in ecosystems as crucial parasitic and predatory natural enemies and are essential for pest control in agricultural and natural ecosystems [148,149,150,151]. Bai et al. [110] reported the presence of 28 spider species from 12 families and 21 genera in the cotton fields of Alaer, Xinjiang, with population dynamics exhibiting three peaks throughout the year, occurring in early July, early August, and early September, which showed a positive correlation with the decline of *L. pratensis* populations, particularly with the populations of *Misumenops tricuspidatus* (Hebei) (Araneae: Thomisidae), *Hylyphantes graminicola* (Sundeval) (Araneae: Linyphiidae), and *Neoscone doenitzi* (Boes. et Str.) (Araneae: Araneidae). These species, which occur in large numbers in cotton fields, are dominant and should be conserved and utilised. Haye [152] identified 65 species of parasitoid wasps from the rearing of *L. pratensis*, with *Peristenus stygicus* (Loan) (Hymenoptera: Braconidae) comprising 52%, *Peristenus digoneutis* (Loan) (Hymenoptera: Braconidae) 34%, and *Mesochorus curvulus* (Thomson) (Hymenoptera: Ichneumonidae) 14%. Laboratory studies found that *P. digoneutis* and *P. stygicus* lay eggs on *L. pratensis*, with an average survival of 25 and 32 days and *P. stygicus* at 782 and *P. digoneutis* at 385. *P. stygicus* has high reproductive potential, ideal field release potential, and cold tolerance, and *P. stygicus* has successfully reduced the density of *Peristenus digoneutis* (Hymenoptera: Braconidae) on alfalfa in the United States, further highlighting the potential of *P. stygicus* for biological control of *L. pratensis* [153]. Chen et al. [154] found that *Metarhizium robertsii* (Metchnikoff) Sorokin (Clavicipitaceae: Metarhizium) kills *Lygus* pests even at temperatures exceeding 35 °C; this fungus has the potential to be used as a biocide to control *Lygus* under high-temperature conditions. Some strains of the entomopathogenic fungus *Beauveria bassiana* (Balsamo-Crivelli) Vuillemin (Cordycipitaceae: Beauveria) reduce *Lygus* population densities in the field [155], providing valuable insights for the biological control of *L. pratensis*.

## 6. Conclusions and Prospects

### 6.1. Conclusions

This paper reviews the recent research on *L. pratensis* and agrees that the surrounding environment significantly influences the occurrence of damage and the control of *L. pratensis*. Non-crop habitats in agricultural landscapes can serve as habitats, breeding sites, and overwintering shelters for *L. pratensis*, where high-density populations can be established before they enter cotton fields. The complexity of controlling *L. pratensis* in cotton fields is further exacerbated by the development of resistance to multiple insecticides. Practices that reduce early insect sources of *L. pratensis* and delay their entry into cotton fields can mitigate the impact of *L. pratensis* on yields and decrease the number of insecticides required. These practices include early spring control of overwintering sites, trapping adults, planting trap crops, managing nitrogen fertiliser applications, and irrigating only when necessary. Although integrated pest management (IPM) methods are employed, foliar insecticides remain the most crucial control measure. New insecticides with unique modes of action are needed for resistance management; concurrently, there is an urgent need to explore effective and sustainable alternatives to the chemical control of *L. pratensis*. This review of the research on *L. pratensis* significantly enhances our understanding of the biology, ecology, and ecological regulation of *L. pratensis*, which can be effectively utilised to develop new control strategies and techniques for the integrated management of *L. pratensis*.

### 6.2. Prospects

#### 6.2.1. Ecological Effects of Agricultural Landscapes

Along with the current research on the ecological effects of agricultural landscapes on the population size of *L. pratensis*, future research is necessary to investigate the conservation efficacy of various types of non-crop habitats on the diversity of natural enemies and the effects on the trophic cascades involving *L. pratensis* and its natural enemies. To avoid inducing population growth of *L. pratensis*, examining the relationship between the biological characteristics of different types of plants in non-crop habitats and their functions in providing food sources and habitats for natural enemies is essential. To encompass the natural enemies of *L. pratensis*, it is advisable to employ traditional species morphological classification and DNA barcode identification techniques in basic research on on-farm habitat management as well as to utilise specific PCR assays to study the predation or parasitism relationships involving the natural enemy of *L. pratensis*, along with controlling the occurrence of *L. pratensis* through interactions within the natural enemy food web. Furthermore, the role of non-crop habitats within agricultural systems, particularly regarding their area share and configuration in enhancing natural enemy diversity and controlling pest populations, warrants thorough investigation. Significant interspecies variations exist in the habits and dispersal abilities of natural enemies, and it is crucial to clarify the migration and dispersal patterns of these natural enemies between non-crop and crop habitats to provide a comprehensive understanding of the mechanisms involved in on-farm habitat management for the ecological regulation of pests.

#### 6.2.2. Development of New Biological Control Methods

Recently, more results have been obtained regarding the behavioural responses of *L. pratensis* to plant volatiles, which should be incorporated into the ecological control technology system for *L. pratensis*. Additionally, in-depth research on chemosensory proteins (CSPs) and olfactory receptors is necessary to identify new odour-binding proteins and transcriptomes. At the same time, RNA interference (RNAi)-mediated gene knockout technology should be employed to influence the olfactory and host selection behaviours of *L. pratensis*. Research on biocontrol agents (including parasitoids and predators) and microorganisms must be intensified in biological control. Currently, no effective biocontrol agents are available, and it is advisable to make further efforts in traditional biological control to identify species compatible with the environmental conditions in Xinjiang.

## Figures and Tables

**Table 2:** Occurrence period and main host plants of *Lygus pratensis* in southern Xinjiang, China.

Generations	Nymph	Adult	Main Host Plants		
			Genus	Species	Region of Distribution *
Overwintering generation		Overwintering adults lay eggs on host plants from late March to mid-April	Halogeton	*Halogeton glomeratus* (Bieb) C. A. Mey. [17]	Canada, China, America, United Kingdom, France, India, Sweden, Spain, Mongolia, Russia, Kazakhstan, Uzbekistan, Kyrgyzstan, Tajikistan, Turkmenistan, etc.
			Suaeda	*Suaeda microphylla* (C. A. Mey.) Pall [18]	China, Russia, Iran, Kazakhstan, Uzbekistan, Kyrgyzstan, Tajikistan, and Turkmenistan
			Kochia	*Kochia prostrata* (L.) Schrad. var. prostrate [19]	China, Mongolia, Russia, Kazakhstan, Uzbekistan, Kyrgyzstan, Turkmenistan
			Descurainia	*Descurainia sophia* (L.) Webb ex Prantl [17]	China, France, Mongolia, Russia, Sweden, Kazakhstan, Uzbekistan, Kyrgyzstan, Turkey, Iran, Afghanistan, America, Canada, Europe, India, etc.
			Brassica	*Brassica napus* L. [64]	China, America, Russia, United Kingdom, Denmark, Sweden, Germany, Switzerland, the Netherlands, etc.
			Medicago	*Medicago sativa* L. [17,27]	China, America, France, Belgium, Sweden, Poland, Canada, Russia, Germany, Spain, Ukraine, Switzerland, the Netherlands, Pakistan, India, etc.
			Triticum	*Triticum aestivum* L. [18]	Every country in the world
			Cirsium	*Cirsium arvense* var. integrifolium C. Wimm. [17]	China, Russia, America, United Kingdom, Germany, Romania, Kazakhstan, Denmark, etc.
First generation	Early to mid-May	Late May to mid-June	Chenopodium	*Chenopodium glaucum* L. [64]	China, America, Canada, United Kingdom, Australia, Sweden, Russia, Slovakia, etc.
			Amaranthus	*Amaranthus retroflexus* L. [18]	China, America, Canada, United Kingdom, Belgium, France, Sweden, Poland, Germany, Spain, India, etc.
			Carthamus	*Carthamus tinctorius* L. [17,27]	China, America, France, United Kingdom, Japan, Sweden, India, Canada, Mexico, Uzbekistan, Slovakia, Slovakia, Greece, etc.
			Kochia	*Kochia prostrata* (L.) Schrad. var. prostrate [19]	China, Mongolia, Russia, Kazakhstan, Uzbekistan, Kyrgyzstan, and Turkmenistan
			Salsola	*Salsola collina* Pall. [17]	China, America, Russia, Poland, Czechia, Canada, Estonia, Latvia, Slovakia, Romania, Belarus, etc.
			Bassia	*Bassia hyssopifolia* (Pall.) O. Kuntze [17]	China, America, Russia, Canada, Belgium, France, Australia, Denmark, Spain, Lithuania, etc.
			Ziziphus	*Ziziphus jujuba* Mill. [18]	China, America, Russia, Australia, Japan, India, Italy, Croatia, Bulgaria, Libya, Romania, Türkiye, etc.
			Brassica	*Brassica napus* L. [27,64]	China, America, Russia, United Kingdom, Denmark, Sweden, Germany, Switzerland, the Netherlands, etc.
			Helianthus	*Helianthus annuus* L. [17,27]	Every country in the world
			Malus	*Malus pumila* Mill. [17]	Asia, Europe, parts of North and South America, and Africa
			Pyrus	*Pyrus brestschneideri* Rehd. [18]	China and Pakistan
Second generation	Mid-late June to early July	Mid- to end of July	Gossypium	*Gossypium herbaceum* L. [64]	China, Brazil, United States, Australia, India, Uzbekistan, Egypt, etc.
Third generation	Early August	Late August	Gossypium	*Gossypium herbaceum* L. [64]	China, Brazil, United States, Australia, India, Uzbekistan, Egypt, etc.
Fourth generation	Mid-September	Late September to mid-late October	Chenopodium	*Chenopodium glaucum* L. [17]	China, America, Canada, United Kingdom, Australia, Sweden, Russia, Slovakia, etc.
			Kochia	*Kochia prostrata* (L.) Schrad. var. prostrate [19]	China, Mongolia, Russia, Kazakhstan, Uzbekistan, Kyrgyzstan, Turkmenistan
			Bassia	*Bassia hyssopifolia* (Pall.) O. Kuntze [18]	China, America, Russia, Canada, Belgium, France, Australia, Denmark, Spain, Lithuania, etc.
			Salsola	*Salsola collina* Pall. [17]	China, America, Russia, Poland, Czechia, Canada, Estonia, Latvia, Slovakia, Romania, Belarus, etc.
			Medicago	*Medicago sativa* L. [17]	China, America, France, Belgium, Sweden, Poland, Canada, Russia, Germany, Spain, Ukraine, Switzerland, the Netherlands, Pakistan, India, etc.

* The region of distribution of all host plants in the table is derived from GBIF (https://www.gbif.org/, accessed on 28 December 2025).

**Table 3:** Correspondence between plant volatiles and host plants.

Plant Volatiles	Source
α-Pinene	*Chenopodium glaucum* L., *Convolvulus arvensis* L., and *Chenopodium serotinum* L. [19]
1,6,10-Dodecatriene, 7,11-dimethyl-3-methylene, (*E*)-	*Convolvulus arvensis* L. [19]
1-Caryophyllene	*Chenopodium glaucum* L. and *Convolvulus arvensis* L. [19]
3-Hexen-1-ol, acetate, (*Z*)-	*Chenopodium glaucum* L., *Convolvulus arvensis* L., and *Lycopersicon esculentum* Mill [19]
Nonanal	*Kochia prostrata* (L.) Schrad. var. prostrate, *Chenopodium glaucum* L., and *Brassica napus* L. [19,64]
Dibutyl phthalate	*Brassica oleracea* var. *botrytis* L. [19]
1-Hexanol, 2-ethyl	*Kochia prostrata* (L.) Schrad. var. prostrate and *Chenopodium glaucum* L. [19]
Butylated hydroxytoluene	*Brassica oleracea* var. *botrytis* L. [19]
1(3H)-Isobenzofuranone, 6-(dimethylamino)-3,3-bis [4-(dimethylamino) phenyl]	*Kochia prostrata* (L.) Schrad. var. prostrate and *Brassica campestris* L. ssp. *chinensis* Makino [19]
Methyl salicylate	*Solanum nigrum* L. and *Brassica napus* L. [64]
Linalool	*Chenopodium glaucum* L. [64]
2-Hexen-1-ol, (*E*)-	*Gossypium herbaceum* L. [64]
Ocimene	*Gossypium herbaceum* L., *Chenopodium glaucum* L., and *Portulaca oleracea* L. [64]
Isothiocyanic acid sec-butyl ester	*Brassica napus* L. [64]
Phenylacetaldehyde	*Solanum nigrum* L. and *Brassica napus* L. [64]

## Data Availability

The original contributions presented in this study are included in the article. Further inquiries can be directed to the corresponding author.
